# Inflammatory Bowel Disease and Risk of Adverse Pregnancy Outcomes

**DOI:** 10.1371/journal.pone.0129567

**Published:** 2015-06-17

**Authors:** Heather A. Boyd, Saima Basit, Maria C. Harpsøe, Jan Wohlfahrt, Tine Jess

**Affiliations:** Department of Epidemiology Research, Statens Serum Institut, Copenhagen, Denmark; Charité-Universitätsmedizin Berlin, GERMANY

## Abstract

**Background and Objectives:**

Existing data on pregnancy complications in inflammatory bowel disease (IBD) are inconsistent. To address these inconsistencies, we investigated potential associations between IBD, IBD-related medication use during pregnancy, and pregnancy loss, pre-eclampsia, preterm delivery, Apgar score, and congenital abnormalities.

**Methods:**

We conducted a cohort study in >85,000 Danish National Birth Cohort women who were pregnant in the period 1996-2002 and had information on IBD, IBD-related medication use (systemic or local corticosteroids, 5-aminosalicylates), pregnancy outcomes and potential confounders. We evaluated associations between IBD and adverse pregnancy/birth outcomes using Cox regression and log-linear binomial regression.

**Results:**

IBD was strongly and significantly associated with severe pre-eclampsia, preterm premature rupture of membranes and medically indicated preterm delivery in women using systemic corticosteroids during pregnancy (hazard ratios [HRs] >7). IBD was also associated with premature preterm rupture of membranes in women using local corticosteroid medications (HR 3.30, 95% confidence interval [CI] 1.33-8.20) and with medically indicated preterm delivery (HR 1.91, 95% CI 0.99-3.68) in non-medicated women. Furthermore, IBD was associated with low 5-minute Apgar score in term infants (risk ratio [RR] 2.19, 95% CI 1.03-4.66). Finally, Crohn’s disease (but not ulcerative colitis) was associated with major congenital abnormalities in the offspring (RR 1.85, 95% CI 1.06-3.21). No child with a congenital abnormality born to a woman with IBD was exposed to systemic corticosteroids in utero.

**Conclusion:**

Women with IBD are at increased risk of severe pre-eclampsia, medically indicated preterm delivery, preterm premature rupture of membranes, and delivering infants with low Apgar score and major congenital malformations. These associations are only partly explained by severe disease as reflected by systemic corticosteroid use.

## Introduction

In 2010, the European Crohn’s Colitis Organisation issued a consensus report summarizing the existing evidence for links between inflammatory bowel disease (IBD) and adverse reproductive outcomes [1]. According to the report, “data on the frequency of complications of pregnancy and labour are very inconsistent, precluding a meaningful conclusion” [1]. Studies of preterm delivery and low birth weight have yielded the most consistent results, with most studies finding that women with IBD are at increased risk of both outcomes [1,2]. Pregnancy loss, pre-eclampsia, placental problems and most neonatal outcomes have been studied very little [1]. Congenital abnormalities have received much attention but published results diverge greatly [1,2]. Whether disease activity and/or medication use during pregnancy affect the risks of adverse pregnancy outcomes is also unclear. Contributing to the difficulty in addressing these issues are the relatively low prevalence of IBD and the infrequency of outcomes such as congenital abnormalities, which together can limit the power of even the most well-conducted studies (e.g. [3]).

Previously, Ajslev and colleagues studied the link between IBD and low birth weight in the Danish National Birth Cohort (DNBC) [4]. Our study also took advantage of the DNBC’s 700 women diagnosed with IBD before pregnancy and its information on potential confounders and early pregnancy losses. We linked DNBC information and Danish national health register data to investigate potential associations between IBD and pregnancy loss, pre-eclampsia, preterm delivery, Apgar score, and congenital abnormalities, taking IBD-related medication use into account.

## Methods

### Study cohorts

Between 1996 and 2003, the DNBC followed >101,000 pregnancies from first trimester until delivery or adverse pregnancy outcome [5]. Different sets of DNBC women were eligible for inclusion in our study cohort depending on the outcome of interest. In our analysis of pregnancy loss, we included all DNBC women in the study cohort. For analyses of pre-eclampsia and preterm delivery, women with singleton pregnancies lasting ≥126 days and ≥140 days, respectively, were included in the study cohorts, whereas only women delivering live singletons were included in our analyses of Apgar score and congenital abnormalities. Women with ≥2 DNBC pregnancies contributed only their first eligible pregnancy to the relevant study cohort.

### Ascertainment of IBD and IBD-related medication use

Based on discharge diagnoses registered in the Danish Hospital Discharge Register [6] in connection with hospital admissions (1977 onward) and outpatient visits (1995 onward), we identified women diagnosed with Crohn’s disease (CD) or ulcerative colitis (UC) before the end of the study pregnancy (International Classification of Disease, 8^th^ revision [ICD-8], codes: CD, 563.00–563.09; UC, 563.19 and 569.04; 10^th^ revision [ICD-10] codes: CD, K50.0-K50.9; UC, K51.0-K51.9). Women with IBD diagnoses first registered after the study pregnancy were not considered to have IBD during the pregnancy. If both CD and UC were registered before or during the study pregnancy, the most recent diagnosis was used.

Using information from the Danish Register of Medicinal Product Statistics, we determined which women with IBD filled prescriptions for the following medications ≤1 month before the estimated date of conception or during the study pregnancy: 5-aminosalicylates (Anatomical Therapeutic Chemical [ATC] code A07EC); azathioprine (L04AX01); local corticosteroids (A07EA); oral corticosteroids (H02AB). These medications were those most commonly prescribed in Denmark for control of IBD symptoms in the period 1996–2003; biologic therapies were not used in pregnant women in that period. Women who filled a prescription for oral (systemic) corticosteroids (OCSs) were classified as OCS users regardless of any other medication use. Women who filled a prescription for local corticosteroids (LCSs) but did not require OCSs were classified as LCS users. AZA users were defined as women with a record of azathioprine use but no corticosteroid use, while 5-ASA users were defined as women whose only filled prescriptions were for 5-aminosalicylates. Since there were only 6 AZA users, these women were excluded from analyses focused on specific medications.

### Outcome ascertainment

#### Pregnancy loss

Pregnancy loss included spontaneous abortions (miscarriages), missed abortions and stillbirths; elective and therapeutic terminations (including termination of ectopic and molar pregnancies) were not considered losses. Pregnancy losses were identified using DNBC records, which are virtually complete with respect to pregnancy outcome. (Only 67 of 101,033 pregnancies had an unknown outcome.)

#### Pre-eclampsia

Women registered in the Hospital Discharge Register with ICD-10 codes O14.0-O15.9 after 18 weeks gestation were considered to have pre-eclampsia. (Due to the very low incidence of eclampsia [O15], we considered eclampsia and pre-eclampsia together as though the former were simply a particularly severe form of the latter. For simplicity, we refer to the aggregate condition as “pre-eclampsia”.) Severe pre-eclampsia was defined as registration with ICD-10 codes O14.1, O14.2 or O15.0–15.9. Early-onset severe pre-eclampsia was defined as severe pre-eclampsia registered before 34 weeks gestation.

#### Preterm delivery (PTD)

We defined PTD as delivery of a living singleton at <37 weeks gestation and classified PTDs as preterm premature rupture of membranes (PPROM), medically-indicated, complication-related or spontaneous. We identified PPROM and medically indicated PTDs using the Danish Medical Birth Register [7]. We included as medically-indicated PTDs all non-PPROM PTDs with record of an induction or a Caesarean section before spontaneous onset of labor. We classified PTD as complication-related if there was no record of an induction or a Caesarean section, but the woman was registered in either the Medical Birth Register or the Hospital Discharge Register with a complication predisposing to PTD (S1 File) or a major offspring birth defect (S2 File) was registered in the Hospital Discharge Register or reported by the mother to the DNBC. PTDs not falling in any of the above three categories were considered to be spontaneous preterm labor without membrane rupture (spontaneous PTD).

#### Apgar score

We obtained 5-minute Apgar scores for the offspring from the Medical Birth Register. Scores <7 were considered low, while scores ≥7 were considered normal/acceptable.

#### Congenital abnormalities

We identified offspring with congenital abnormalities using the Hospital Discharge Register. The designation “congenital abnormalities overall” included all registered abnormalities, no matter how minor (ICD-10 codes Q00.0-Q99.9). We defined major congenital abnormalities as defects considered major by either the Metropolitan Atlanta Congenital Defects Program (www.cdc.gov/ncbddd/birthdefects/macdp.html) or EUROCAT (www.eurocat-network.eu) (S2 File).

### Ascertainment of potential confounders

We decided a priori to adjust all estimates for the woman’s age, socioeconomic status (SES), parity, pre-pregnancy BMI, smoking and alcohol consumption, as these variables may affect the risk of pregnancy and birth outcomes and are also associated with IBD. Information on parity was obtained from the Medical Birth Register. Information on the remaining variables was obtained from DNBC prenatal interviews conducted at ca. 16 and 30 weeks gestation. Our SES variable combined information on educational attainment and current employment for the adult in the household with the highest SES at the beginning of the pregnancy. The smoking and alcohol variables combined information from the two interviews and represented the woman’s behavior while pregnant; pre-pregnancy information was not available for smoking.

Both inadequate periconceptional folic acid levels and parental congenital abnormalities have been associated with congenital abnormalities in the offspring. While it seemed unlikely that either folic acid use or parental birth defects would also be associated with maternal IBD, we investigated whether periconceptional folic acid use and/or major maternal congenital abnormalities might confound associations between maternal IBD and offspring congenital abnormalities. Information on folic acid use in the period from 4 weeks before conception to 8 weeks after conception was obtained from the first DNBC prenatal interview. Information on major maternal congenital abnormalities (considered as a group, due to their rarity) was obtained from the Hospital Discharge Register using the same definition as for major abnormalities in the offspring.

### Follow-up in the adverse pregnancy event cohorts

#### Pregnancy loss

Women were followed from estimated date of conception until the first of the following events: 1) miscarriage/stillbirth; 2) elective or therapeutic termination of pregnancy; 3) death or emigration while still pregnant; 4) live birth; or 5) 42 weeks gestation.

#### Pre-eclampsia

Women were followed from the 126^th^ day of pregnancy (18 completed weeks) until the first of the following: 1) pre-eclampsia; 2) emigration; or 3) the end of pregnancy (termination, loss, delivery or maternal death).

#### Preterm delivery

Women were followed from the 140^th^ day of pregnancy (20 completed weeks) until the first of the following events: 1) PTD; 2) late termination of pregnancy; 3) stillbirth; 4) maternal death or emigration at <259 days gestation; or 5) 259 days gestation (37 completed weeks, no longer at risk of PTD).

### Statistical analyses

#### Adverse pregnancy events

We used Cox proportional hazards modelling with age as the underlying timescale to estimate hazard ratios comparing rates of pregnancy loss, pre-eclampsia and PTD in women with and without IBD, overall and separately for CD and UC. All hazard ratios were adjusted for parity, SES, pre-pregnancy BMI, and smoking and alcohol consumption during the study pregnancy in strata within the models. PTD subtype analyses used competing risk methodology, whereby women were censored at delivery if they had a PTD of a type other than the subtype of interest. We tested for differences in estimates for CD and UC using Wald Chi-square tests. The proportional hazards assumption was evaluated using a time-dependent IBD variable.

#### Birth outcomes

We used log-linear binomial regression to estimate risk ratios comparing risk of low Apgar score and congenital abnormalities in the offspring of women with and without IBD, overall and separately for CD and UC, adjusting for maternal age, parity, SES, pre-pregnancy BMI, and smoking and alcohol consumption during the study pregnancy. Risk ratios for congenital abnormalities were also adjusted for periconceptional folic acid use; we also investigated the effect of adjusting for major maternal congenital abnormalities.

### Ethics

The Department of Epidemiology Research, Statens Serum Institut, is authorized by the Danish Data Protection Agency (Datatilsynet) to conduct register-based studies (approval number 2008-54-0472). Our study was registered with the Data Protection Agency in accordance with this agreement. The Data Protection Agency (approval number 2012-54-0268) and the Danish Capital City Region's Committee for Biomedical Research Ethics (approval number (KF) 01-471/94) previously approved the DNBC’s establishment and its use for research purposes. According to Danish law (and the aforementioned approvals), we were not required to obtain informed consent from DNBC women to use their register information. All data were anonymized and de-identified prior to analysis.

## Results

### Pregnancy loss

The pregnancy loss cohort included 91,700 women, 700 of whom were diagnosed with IBD before the end of the study pregnancy (CD, 295; UC, 405). The pregnancy ended in miscarriage for 4,447 women without IBD (4.9%), 16 with CD (5.4%) and 15 with UC (3.7%), and stillbirth for 573 non-IBD women (0.6%), 1 with CD (0.3%) and 3 with UC (0.7%); 594 women terminated their pregnancy. Maternal IBD was not associated with an increased rate of pregnancy loss (hazard ratio [HR] 0.75, 95% confidence interval [CI] 0.33–1.69; CD: HR 0.91, 95% CI 0.28–2.88; UC: HR 0.64, 95% CI 0.20–2.00). Relaxing our definition of an IBD-exposed pregnancy to include pregnancies in women diagnosed with IBD after the study pregnancy (up to December 2011) did not change our results appreciably (S3 File).

### Pre-eclampsia

The pre-eclampsia cohort comprised 86,792 women, including 666 diagnosed with IBD before the end of the study pregnancy (CD, 278; UC, 388). The overall pre-eclampsia rate in women with IBD did not differ significantly from that in women without IBD (HR 1.21, 95% CI 0.76–1.95) (Table 1). However, rates of severe pre-eclampsia were more than 2-fold greater in women with IBD than in IBD-free women (Table 1). Estimates for CD and UC were not statistically significantly different from one another (p_severe pre-eclampsia_ = 0.07, p_early-onset severe pre-eclampsia_ = 0.13).

**Table 1:** Associations between inflammatory bowel disease, medication use and rates of pre-eclampsia in the Danish National Birth Cohort, 1996–2003

	No pre-eclampsia	Any pre-eclampsia^a^			Severe pre-eclampsia^b^			Early-onset severe pre-eclampsia^c^		
	n	n	HR	95% CI	n	HR	95% CI	n	HR	95% CI
Inflammatory bowel disease										
Yes	644	22	1.21	0.76–1.95	8	2.24	1.05–4.80	5	2.72	1.00–7.35
Systemic steroid use	39	6	3.52	1.36–9.13	4	17.4	3.72–81.4	3	21.6	3.42–136
No systemic steroid use^d^	599	16	0.95	0.54–1.67	4	1.30	0.47–3.60	2	1.31	0.31–5.57
No	83,795	2,331	Ref		459	Ref		226	Ref	
Crohn’s disease										
Yes	266	12	1.42	0.71–2.86	5	2.96	1.09–8.03	2	2.18	0.42–11.4
Systemic steroid use	15	4	4.87	1.25–19.0	3	56.8	3.53–914	2	50.1^e^	11.9–210
No systemic steroid use^d^	245	8	0.98	0.40–2.41	2	1.51	0.36–6.30	0	-	-
No	83,795	2,331	Ref		459	Ref		226	Ref	
Ulcerative colitis										
Yes	378	10	1.07	0.56–2.05	3	1.63	0.49–5.37	3	3.12	0.91–10.7
Systemic steroid use	24	2	2.57	0.60–11.0	1	8.38	0.88–80.1	1	14.0 ^e^	1.95–101
No systemic steroid use^d^	354	8	0.93	0.45–1.91	2	1.14	0.27–4.83	2	2.33 ^e^	0.58–9.39
No	83,795	2,331	Ref		459	Ref		226	Ref	

HR, hazard ratio; CI, confidence interval.

All estimates were adjusted for parity (0, 1, ≥2), socioeconomic status (6 categories: master’s degree or higher and currently employed, or leader of a business with ≥10 employees; bachelor’s degree and currently employed, or leader of a business with <10 employees; skilled worker (completed vocational training with apprenticeship) and currently employed; unskilled worker or unemployed (short-term); current student; unemployed (long-term)), pre-pregnancy BMI (<20, 20–25, >25), and smoking (non-smoker, smoker) and alcohol consumption (non-drinker, <1 drink/week, ≥1 drink/week) during pregnancy, in strata within the models.

^a^ ICD-10 codes O14.0-O15.9 registered in the Hospital Discharge Register.

^b^ ICD-10 codes O14.1, O14.2 or O15.0–15.9 registered in the Hospital Discharge Register.

^c^ ICD-10 codes O14.1, O14.2 or O15.0–15.9 registered in the Hospital Discharge Register before 34 weeks’ gestation.

^d^ Includes women with IBD who used internal corticosteroids or 5-ASA during pregnancy, as well as women who used no medication.

^e^ When we modelled the effect of IBD type and medication use on the rate of severe, early pre-eclampsia with the covariates in strata, the models did not converge. Consequently, the estimates presented here were adjusted for the potential confounders through inclusion of the covariates as independent variables in the model.

When we considered IBD status in combination with medication use, our numbers were sufficient only to differentiate between users and non-users of OCSs. Our results suggested that the increased rates of severe pre-eclampsia associated with IBD were driven by tremendous increases in rates among OCS users, whereas there was little suggestion of an increase in pre-eclampsia rates in women with IBD who did not use OCSs while pregnant (Table 1).

### Preterm delivery

There were 86,591 women included in the PTD cohort, including 666 diagnosed with IBD before the end of the study pregnancy (CD, 278; UC, 388). Overall, women with IBD had rates of PTD that were 2-fold greater than those observed in women without IBD (HR 2.20, 95% CI 1.71–2.84). This association appeared stronger for UC than for CD (Table 2), but the estimates were not significantly different from one another (p = 0.29). Women with IBD who used OCSs during pregnancy were at particularly high risk of delivering prematurely (HR 6.32, 95% CI 3.13–12.7) (Table 2); interestingly, 13 of the 14 OCS-using women with IBD who delivered preterm had disease activity registered during pregnancy. Women who used other IBD medications during pregnancy also had increased rates of preterm delivery, although to a much more modest extent (<80% increase in rates compared with women without IBD, Table 2), and even women who did not use medication while pregnant had moderately increased rates of PTD (Table 2).

**Table 2:** Associations between inflammatory bowel disease, medication use during pregnancy and overall rates of preterm delivery in the Danish National Birth Cohort, 1996–2003.

	Term delivery	Preterm delivery^a^		
	n	n	HR	95% CI
Inflammatory bowel disease				
Yes	603^b^	63	1.97	1.46–2.64
Systemic steroid use	31	14^c^	6.32	3.13–12.7
Internal steroid use	63	7	1.64	0.60–4.47
5-ASA use	167	15	1.77	1.01–3.13
No medication use	336	27	1.53	0.98–2.41
No	82,040	3,885	Ref	
Crohn’s disease				
Yes	253 ^b^	25	1.61	0.99–2.62
Systemic steroid use	13	6	3.56	1.18–10.8
Internal steroid use	7	2	6.31	0.80–50.1
5-ASA use	50	3	0.94	0.23–3.88
No medication use	177	14	1.39	0.74–2.62
No	82,040	3,885	Ref	
Ulcerative colitis				
Yes	350	38	2.24	1.55–3.24
Systemic steroid use	18	8	9.98	4.04–24.6
Internal steroid use	56	5	1.32	0.43–4.04
5-ASA use	117	12	2.12	1.13–3.95
No medication use	159	13	1.71	0.90–3.23
No	82,040	3,885	Ref	

HR, hazard ratio; CI, confidence interval.

All estimates were adjusted for parity (0, 1, ≥2), socioeconomic status (6 categories: master’s degree or higher and currently employed, or leader of a business with ≥10 employees; bachelor’s degree and currently employed, or leader of a business with <10 employees; skilled worker (completed vocational training with apprenticeship) and currently employed; unskilled worker or unemployed (short-term); current student; unemployed (long-term)), pre-pregnancy BMI (<20, 20–25, >25), and smoking (non-smoker, smoker) and alcohol consumption (non-drinker, <1 drink/week, ≥1 drink/week) during pregnancy, in strata within the models.

^a^ Delivery before 37 weeks’ gestation.

^b^ The medication use sub-categories do not sum to the total number of women with IBD and CD because 6 women with CD who used AZA were excluded from the medication type-specific analyses.

^c^ Registered pregnancy complications and other potentially relevant conditions in OCS-using women with IBD who delivered prematurely: preterm premature rupture of membrances (five women); severe pre-eclampsia (four women); maternal chronic disease other than IBD (four women, one with spontaneous preterm delivery and three with indicated Caesarian deliveries; the degree to which these conditions, rather than IBD activity, influenced the decision to deliver the fetus early in the medically indicated deliveries, is unclear); breech presentation of fetus (one woman with medically indicated preterm delivery).

When we examined PTD subtypes, we found that IBD was associated with a 2-fold increase in the rate of PPROM, an increase that was driven by women with UC, as very few women had both CD and PPROM (Table 3). Women with IBD who used OCSs while pregnant had a 24-fold increase in the rate of PPROM compared with women without IBD, while pregnant women with IBD using other medications had more than triple the PPROM rate (Table 3).

**Table 3:** Associations between inflammatory bowel disease, medication use during pregnancy and rates of preterm delivery (PTD) subtypes in the Danish National Birth Cohort, 1996–2003.

	PPROM			Medically-indicated PTD			Spontaneous PTD		
	n	HR	95% CI	n	HR	95% CI	n	HR	95% CI
Inflammatory bowel disease									
Yes	12	2.28	1.18–4.39	29	2.18	1.38–3.46	19	1.50	0.89–2.53
Systemic steroid use	5	24.0	6.28–91.5	7	7.54	2.51–22.6	1	0.88	0.11–7.00
Other medication use^a^	7	3.30	1.33–8.20	8	1.60	0.69–3.69	7	1.40	0.60–3.26
No medication use	0	-	-	14	1.91	0.99–3.68	11	1.71	0.85–3.43
No	806	Ref		1,281	Ref		1,599	Ref	
Crohn’s disease									
Yes	2	0.64	0.09–4.83	14	2.22	1.09–4.52	7	1.17	0.50–2.72
Systemic steroid use	0	-	-	5	6.43	1.60–25.8	1	1.73	0.19–15.5
Other medication use^a^	2	4.17	0.47–37.0	1	1.38	0.18–10.5	2	0.90	0.12–6.63
No medication use	0	-	-	8	1.70	0.64–4.50	4	1.17	0.42–3.31
No	794	Ref		1,252	Ref		1,599	Ref	
Ulcerative colitis									
Yes	10	3.08	1.52–6.22	15	2.15	1.18–3.93	12	1.80	0.93–3.50
Systemic steroid use	5	38.0	8.29–174	2	9.84	1.69–57.2	0	-	-
Other medication use^a^	5	3.17	1.17–8.61	7	1.65	0.65–4.15	5	1.59	0.62–4.05
No medication use	0	-	-	6	2.11	0.87–5.16	7	2.61	1.02–6.69
No	794	Ref		1,252	Ref		1,599	Ref	

HR, hazard ratio; CI, confidence interval; PTD, preterm delivery; PPROM, premature preterm rupture of membranes.

All estimates were adjusted for parity (0, 1, ≥2), socioeconomic status (6 categories: master’s degree or higher and currently employed, or leader of a business with ≥10 employees; bachelor’s degree and currently employed, or leader of a business with <10 employees; skilled worker (completed vocational training with apprenticeship) and currently employed; unskilled worker or unemployed (short-term); current student; unemployed (long-term)), pre-pregnancy BMI (<20, 20–25, >25), and smoking (non-smoker, smoker) and alcohol consumption (non-drinker, <1 drink/week, ≥1 drink/week) during pregnancy, in strata within the models.

^a^ Includes women with IBD who used internal corticosteroids or 5-ASA during pregnancy.

IBD was also associated with a 2-fold increase in the rate of medically-indicated PTD, with estimates for CD and UC having similar magnitudes (Table 3). This association was strongest among OCS users, whose rate of medically-indicated PTD was more than 7 times that of women without IBD (Table 3). However, our results were also consistent with an almost 2-fold increased rate of medically-indicated PTD among women with no registered medication use (Table 3).

Our results also suggested that women with IBD might have a modestly increased risk of spontaneous PTD (Table 3). There were too few women with both medication use during pregnancy and spontaneous PTD to estimate the effect of medication use on the rate of spontaneous PTD with any confidence. However, women with IBD who did not use medication during pregnancy appeared to have a 70% increase in spontaneous PTD rate, compared with women without IBD; for women with UC, the spontaneous PTD rate increased 2.6-fold (Table 3).

There were too few women (n = 243) with complication-related PTD to allow for meaningful analyses for this PTD subtype.

### Birth outcomes

The Apgar score cohort included 85,445 women, 654 of whom were diagnosed with IBD before the end of the study pregnancy (CD, 276; UC, 378). Among the cohort women, 629 (0.7%) delivered a living singleton whose 5-minute Apgar score was <7. Women with IBD were more than 1.5 times as likely as women without IBD to deliver a child with a low Apgar score, although this increase in risk was not statistically significant (Table 4). Restricting our analyses to women who delivered their babies at ≥37 weeks gestation (since the Apgar score is of limited use in preterm infants [8]) yielded a stronger, statistically significant association between IBD and low Apgar score (Table 4). Women with CD had a more than 3-fold increased risk of delivering a child with a low Apgar score, whereas there appeared to be no association between UC and Apgar score (Table 4); however, the two estimates were not significantly different (p = 0.17). Since there were so few women with IBD who delivered infants with low Apgar scores, we could not assess the impact of IBD medication use on the risk of low Apgar score.

**Table 4:** Associations between inflammatory bowel disease and risks of low 5-minute Apgar score and congenital abnormalities in living singletons born to Danish National Birth Cohort mothers, 1996–2003.

	5-minute Apgar score														
	All infants				Term infants only				No cong.	Any congenital			Major^a^ congenital		
	Apgar≥7	Apgar<7			Apgar≥7	Apgar<7			abnorm.	abnormality			abnormality		
	n	n	RR	95% CI	n	n	RR	95% CI	n	n	RR	95% CI	n	RR	95% CI
Inflammatory bowel disease															
Yes	646	8	1.62	0.77–3.40	588	8	2.19	1.03–4.66	609	54	1.09	0.82–1.44	19	1.20	0.76–1.89
No	84,170	621	Ref		80,696	488	Ref		79,545	6,013	Ref		2,286	Ref	
Crohn’s disease															
Yes	271	5	2.63	1.10–6.28	249	5	3.55	1.45–8.67	255	24	1.22	0.82–1.82	12	1.85	1.06–3.21
No	84,170	621	Ref		80,696	488	Ref		79,545	6,013	Ref		2,286	Ref	
Ulcerative colitis															
Yes	375	3	0.83	0.21–3.31	339	3	1.12	0.28–4.53	354	30	0.99	0.66–1.46	7	0.71	0.32–1.56
No	84,170	621	Ref		80,696	488	Ref		79,545	6,013	Ref		2,286	Ref	

RR, risk ratio; CI, confidence interval.

All estimates were adjusted for maternal age (≤25, 26–30, 31–35, >35 years), parity (0, 1, ≥2), socioeconomic status (6 categories: master’s degree or higher and currently employed, or leader of a business with ≥10 employees; bachelor’s degree and currently employed, or leader of a business with <10 employees; skilled worker (completed vocational training with apprenticeship) and currently employed; unskilled worker or unemployed (short-term); current student; unemployed (long-term)), pre-pregnancy BMI (<20, 20–25, >25), and smoking (non-smoker, smoker) and alcohol consumption (non-drinker, <1 drink/week, ≥1 drink/week) during pregnancy. The congenital abnormality estimates were additionally adjusted for maternal folic acid use in the period from 4 weeks before conception to 8 weeks after conception (any use/no use).

^a^ Congenital abnormalities defined as major by the Metropolitan Atlanta Congenital Defects Program and/or EUROCAT. See Supplemental Digital Content for a list of conditions included.

The congenital abnormalities cohort included 86,221 women, 663 with an IBD diagnosis registered before the end of the study pregnancy (CD, 279; UC, 384). Of these women, 6,067 (7.0%) delivered a child with a congenital abnormality; 2,305 (2.7%) of the offspring had a major abnormality. The risk of delivering a living singleton with a congenital abnormality did not differ for women with and without IBD, both overall and for major abnormalities alone (Table 4). However, women with CD had a moderately increased risk of delivering a child with a major congenital abnormality (RR 1.85, 95% CI 1.06–3.21). In contrast, our findings did not support an increased risk of major congenital abnormalities in the offspring associated with UC (RR 0.71, 95% CI 0.32–1.56; p_CD vs. UC_ = 0.05). The same pattern was observed when we adjusted for major maternal congenital abnormalities (along with the potential confounders identified a priori) instead of periconceptional folic acid use (RR_IBD_ 1.17, 95% CI 0.75–1.83; RR_CD_ 1.72, 95% CI 0.99–2.99; RR_UC_ 0.76, 95% CI 0.36–1.58). (Adjustment for maternal abnormalities and folic acid use in the same model proved impossible.)

Major abnormalities in the offspring of women with CD included choanal atresia (3 children), congenital hip dislocation (2), spina bifida (2), talipes equinovarus (2), cleft lip and palate (1), cystic kidney disease (in a child with talipes), hydrocephalus (in a child with choanal atresia), hypospadias (1), and polydactyly (1). Major abnormalities in the offspring of women with UC included congenital hip dislocation (3), cleft palate ± cleft lip (2), Hirschsprung disease (1) and talipes calcaneovarus (1). When we looked at specific major abnormalities affecting ≥2 children born to IBD mothers, we found that CD was very strongly associated with spina bifida (RR 20.2, 95% CI 4.80–84.9) and choanal atresia (RR 125, 95% CI 32.7–478). Confidence intervals for cleft palate ± cleft lip, congenital hip dislocation/subluxation and talipes were too wide to allow us to draw meaningful conclusions (S1 Table).

Of the 19 children with major abnormalities born to women with IBD, seven were exposed to medication in utero. No child was exposed to OCSs; the child with talipes born to a woman with UC was exposed to LCSs. Six children were born to women who took 5-ASA while pregnant (3 children with congenital hip dislocation, and 1 each with cleft palate, hypospadias and polydactyly).

## Discussion

We investigated associations between IBD, IBD-specific medication use and adverse pregnancy and birth outcomes in the Danish National Birth Cohort. We found no evidence of an association between IBD and pregnancy loss. In contrast, IBD was strongly associated with severe pre-eclampsia, medically-indicated PTD, PPROM and low 5-minute Apgar score, and modestly associated with spontaneous PTD. CD was also moderately associated with delivering a child with a major congenital abnormality.

When we examined the effect of medication use for IBD, it became clear that the observed increase in risk of severe pre-eclampsia associated with IBD was limited to women who used OCSs during pregnancy, and that the associations with PPROM and medically-indicated PTD were also largely driven by OCS users. However, we also found associations with PPROM in users of medications other than OCSs, and possibly between untreated IBD and spontaneous PTD. Women with IBD and no medication use during pregnancy also appeared to have an increased risk of medically-indicated PTD. Furthermore, of the women with IBD whose children had a major congenital abnormality, only 37% had used medication while pregnant, and none was an OCS user.

We found no excess of pregnancy losses among women with IBD, which is consistent with the findings of Stephansson et al. and Cornish et al. for stillbirth and of Bortoli et al. for miscarriage [3,9,10]. An important caveat, however, is that we can say nothing about very early pregnancy losses in women with IBD. Women were recruited into the DNBC between weeks 6 and 12, when they visited their general practitioner to confirm their pregnancy. Women suffering losses occurring before this visit were therefore never included in the cohort. On the other hand, although we had limited power to definitively state that IBD does not increase the pregnancy loss rate and to examine the impact of IBD medication use on pregnancy loss, our results for later losses (miscarriage in physician-confirmed pregnancies and stillbirth) are reassuring nonetheless.

Our pre-eclampsia findings are inconsistent with the results of two previous studies showing no association between IBD [11] or CD [10] and pre-eclampsia. However, Bush et al. included only 116 women with IBD in their study and the determination of no association was made using a simple χ^2^ test [11]. While the study by Stephansson et al. was considerably better powered, they only considered pre-eclampsia in the aggregate (any severity and timing of onset) [10], which is problematic since pre-eclampsia is not a single entity but a syndrome encompassing multiple conditions with similar symptoms. Moderate pre-eclampsia is attributed predominantly to maternal factors such as obesity while severe preeclampsia is characterized by placental pathology, endothelial dysfunction and inflammation. Therefore, considering pre-eclampsia as a single entity could mask associations with pre-eclampsia subtypes, particularly since moderate pre-eclampsia occurs more frequently than severe pre-eclampsia. Indeed, we found no association between IBD and pre-eclampsia overall; however, we observed a strong association between IBD and severe pre-eclampsia. Active inflammatory processes in women with IBD may interfere with proper placentation or with the placenta’s ability to meet increasing fetal demands, leading to severe pre-eclampsia as early as mid-second trimester. Our finding that the increased risk of pre-eclampsia appeared to be limited to OCS users is consistent with this hypothesis, since only women with severe, active IBD are treated with OCSs while pregnant. Consequently, in general, women with IBD need not worry about severe pre-eclampsia. Furthermore, even women requiring treatment with OCSs while pregnant—less than 7% of the women with IBD in our study—need not be unduly concerned: although the risk of severe pre-eclampsia in OCS-using women with IBD was 17 times that of women without IBD, the absolute number of affected women was small (n = 4, 8.8% of OCS-using women), since severe pre-eclampsia occurs infrequently in the general pregnant population. While a recommendation that women with IBD who require systemic steroids while pregnant should be followed more closely than the average pregnant woman for signs of pre-eclampsia seems warranted, women with IBD disease activity and/or those using steroids while pregnant (for whatever reason) are likely already subject to intense medical supervision.

Most [9,10], but not all [3,12], previous studies have found an association between IBD and PTD, typically reporting up to a doubling of PTD risk. However, whether the association only exists for women with active disease/using IBD medications—as has been suggested [1,2,11,13,14]—is unclear. Furthermore, despite many studies showing that women with IBD have increased rates of Caesarian section and induction [1], only one study has looked specifically at rates of medically indicated PTD and other PTD subtypes; Stephansson et al. found a strong association between CD and induced PTD (prevalence odds ratio [POR] = 2.59) and a moderate association (POR = 1.52) with spontaneous PTD [10].

Our spontaneous PTD findings were of similar magnitude to those reported by Stephansson et al. [10], but were driven by women with UC and no medication use. Many of the biologic mechanisms that are upregulated or altered when parturition is initiated (e.g. Th1/Th2 balance, specific cytokine cascades, expression of toll-like receptors, NOD-like receptors and prostaglandins) are also dysregulated in IBD, suggesting that the mechanisms producing IBD pathology can contribute to spontaneous PTD, even in women with quiescent disease [15].

Our findings with respect to medically indicated PTD were consistent with those of Stephansson et al. [10], but were of greater magnitude and applied to both UC and CD. The associations were strongest in women using OCSs while pregnant and were almost certainly driven at least in part by severe pre-eclampsia, which in our cohort was strongly associated with OCS use in women with IBD and which often necessitates early delivery. However, OCS use while pregnant is also a sign of severe, active IBD, which can itself be an indication for early delivery if the condition of mother or fetus warrants it [1,2].

Our finding of strong associations between IBD and PPROM in both women who used OCSs while pregnant and women who used other medications are novel and biologically plausible. Expansion of the fetal membranes as pregnancy progresses and membrane weakening as labor approaches are controlled processes mediated by matrix metalloproteinases (MMPs) and caspases [16]. Systemic inflammation may promote premature upregulation of MMP and caspase activity via proinflammatory cytokine cascades, leading to excessive collagen turnover, extracellular matrix degradation and premature membrane rupture. Medication use during pregnancy, OCS use in particular, in women with IBD is an indicator of active disease with upregulation of inflammatory pathways, potentially including MMPs and caspase. On the other hand, glucocorticoids, the corticosteroids most commonly used to treat IBD flare-ups, may themselves contribute to the initiation of parturition by causing upregulation of fetal prostaglandin synthesis; depending on the timing of exposure, glucocorticoid use may therefore contribute directly to PTD, PPROM in particular [15].

With the exception of low birth weight and possibly congenital abnormalities, the term offspring of women with and without IBD are thought to have similar birth outcomes [1]. However, contrary to the results of Stephansson et al. [10], we found a strong association between IBD and low 5-minute Apgar score in term infants, suggesting that some infants born to women with IBD may be in greater distress, at least in the period immediately after birth, than their counterparts with IBD-free mothers. Whether this is a consequence of pregnancy complications (e.g. preeclampsia), delivery complications, fetal morbidity or some combination of the three needs to be investigated further. However, while the association between IBD and low 5-minute Apgar score was strong in relative terms (HR>3), the absolute number of affected infants was small, indicating that while the midwife/physician overseeing an IBD patient’s delivery should be aware of the greater-than-usual risk of fetal distress, the chance that any given delivery will be affected is still slight.

Whether IBD is linked to congenital abnormalities remains controversial despite the large number of studies that have addressed this issue. Studies have shown UC to be strongly associated with congenital abnormalities overall [9,14] and with limb deficiencies, obstructive urinary anomalies and multiple anomalies in particular [17]; however, other studies have shown no association with UC, overall [3,18] or for the specific abnormalities mentioned [18]. Similarly, studies of CD show no association [3], an overall association [9], and an association with a specific abnormality (limb reduction) [10]. The inconsistencies may stem from differences in the abnormalities included for analysis as well as variations in study power; we saw no association between IBD and congenital abnormalities when both minor and major abnormalities were included in the analysis but a moderate association between CD and major congenital abnormalities as a group. We also saw strong links with specific groups of major abnormalities for CD (spina bifida, choanal atresia) but these did not overlap with previously reported links. However, these results should be interpreted with caution. Our defect-specific findings stem from post hoc analyses, rather than a planned, methodical examination of specific groups of abnormalities. Furthermore, the results are based on very small numbers of exposed outcomes, and even if the increases in risk are real, the absolute risks are still very small. Also worthy of note is that OCS use did not appear to be linked with an increased risk of major congenital abnormalities; six of the seven children with major abnormalities born to mothers who used IBD-related medication while pregnant were exposed only to 5-ASA.

One of our study’s great strengths was its use of the Danish National Birth Cohort, with its large number of women with IBD diagnosed prior to the study pregnancy and minimal loss to follow-up. Use of the DNBC also permitted us to adjust our estimates for potential confounders not available to solely register-based studies. Linkage to the Register of Medicinal Product Statistics allowed us to incorporate information on medication use and thereby differentiate between women with active disease and those whose disease was presumably quiescent.

Although our cohort included a large number of women with IBD, the absolute numbers of adverse pregnancy events and adverse birth outcomes among these women were often small. Consequently, we frequently did not have the power to show differences between the experiences of women with CD and those with UC (if such differences exist), despite finding strong, statistically significant associations between IBD overall and many outcomes. Another potential limitation was the use of filled corticosteroid prescriptions as a proxy for disease activity, under the assumption that corticosteroids are prescribed as sparingly as possible during pregnancy. However, this step meant that we are unable to comment on whether the overwhelming associations observed in OCS users reflect a direct adverse effect of corticosteroids themselves or illustrate the consequences of out-of-control inflammation during pregnancy.

Biologic therapy was introduced in Denmark in 1999; none of the women in our study used these medications. In addition, only six women were treated with azathioprine. It might therefore be argued that it could be difficult to generalize some of the study results to current IBD patients. On the other hand, the extent to which these newer therapies are used in pregnant women is likely limited in many settings. (Likewise, the degree to which these therapies are the current standard of care in the general IBD patient population probably also varies widely). In Denmark in the period 1999–2012, only 8% of all IBD patients were treated with tumor necrosis factor-α antagonists [19], and the use among pregnant women is assumed to have been even more limited. Similarly, while 21.9% of all IBD patients 15 years of age or older were treated with azathioprine during the same period [19], current Danish product labelling recommends that azathioprine not be used by pregnant women. We therefore contend that a study of pregnant patients using therapies other than biologics and azathioprine still represents the experience of contemporary pregnant women with IBD. Furthermore, our findings provide an important baseline against which to compare the pregnancy experiences of IBD patients using newer therapies. Such baseline data on pregnancy outcomes in women using older therapies is currently lacking, making it difficult to evaluate whether biologic therapies are better options for pregnant patients.

Despite its potential limitations, our study is one of the hitherto largest to examine associations between IBD and adverse pregnancy and birth outcomes with detailed information on potential lifestyle confounders and information on IBD-related medication use during pregnancy. We showed that IBD was strongly associated with the rates of severe pre-eclampsia, PPROM and medically induced PTD, associations that were largely limited to OCS users. However, additional associations between IBD and PPROM and spontaneous PTD, and between CD and major congenital abnormalities, all among women not using OCSs, suggest that some feature of even quiescent IBD plays a major role in several adverse pregnancy and birth outcomes.

## Supporting Information

S1 File Pregnancy complications predisposing to preterm delivery. (DOCX)

S2 File Major congenital abnormalities. (DOCX)

S3 File Pregnancy loss results including post-pregnancy IBD diagnoses in definition of IBD. (DOCX)

S1 Table Complete results for specific groups of major congenital abnormalities. (DOCX)
