# Supplementary material for: Inflammatory Bowel Disease and Risk of Adverse Pregnancy Outcomes
Source: PLoS One. 2015 Jun 17;10(6):e0129567. doi: 10.1371/journal.pone.0129567 (PMC4471220; doi:10.1371/journal.pone.0129567)
Supplement: S1 File — (DOCX) [file pone.0129567.s001.docx]

**Pregnancy complications predisposing to preterm delivery**

Conditions considered to predispose a woman to preterm delivery, and therefore included in our definition of complication-related preterm delivery if they were registered during a pregnancy that ended in a preterm delivery without induction of labor or Caesarian section: pre-eclampsia/eclampsia, uterine or cervical abnormalities, suspected fetal abnormality, isoimmunization, hydrops fetalis, polyhydramnios, chorioamnionitis, placental abnormalities, placenta previa and placental abruption.
