# Supplementary material for: Inflammatory Bowel Disease and Risk of Adverse Pregnancy Outcomes
Source: PLoS One. 2015 Jun 17;10(6):e0129567. doi: 10.1371/journal.pone.0129567 (PMC4471220; doi:10.1371/journal.pone.0129567)
Supplement: S2 File — (DOCX) [file pone.0129567.s002.docx]

**Major congenital abnormalities**

Congenital abnormalities (with relevant ICD-10 codes) occurring in the Danish National Birth Cohort and considered major for the purposes of our analyses, based on the definitions used by the Metropolitan Atlanta Congenital Defects Program (MACDP) and EUROCAT:

Nervous system abnormalities: Q00.0 (anencephaly), Q01.0-01.9 (encephalocele), Q03.0-03.9 (congenital hydrocephalus), Q05.0-05.9 (spina bifida).

Ocular abnormalities: Q11.0-11.1 (cystic eyeball or other anopthalmos), Q12.0 (congenital cataract).

Cardiac and circulatory system abnormalities: Q20.0 (common arterial trunk), Q20.1 (double outlet right ventricle), Q20.3-20.5 (discordant ventriculoarterial connection, double inlet ventricle, discordant atrioventricular connection), Q21.0 (ventricular septal defect), Q21.2-21.4 (atrioventricular septal defect, Tetralogy of Fallot, aortopulmonary septal defect), Q22.0-22.2 (pulmonary valve atresia, congenital pulmonary valve stenosis or insufficiency), Q22.5 (Ebstein’s anomaly), Q23.0 (congenital aortic valve stenosis), Q23.2 (congenital mitral stenosis), Q23.4 (hypoplastic left heart syndrome), Q25.1 (coarctation of aorta), Q25.4 (other congenital malformations of aorta), Q26.2-26.4 (anomalous pulmonary venous connection, total, partial or unspecified).

Respiratory system abnormalities: Q30.0 (choanal atresia), Q35.0-37.9 (cleft palate, cleft lip, cleft palate with cleft lip).

Digestive system abnormalities: Q39.0-39.4 (esophageal atresia with or without fistula, congenital tracheo-esophageal fistula without atresia, congenital stenosis and stricture of esophagus, esophageal web), Q40.0 (congenital hypertrophic pyloric stenosis), Q41.0-41.2, 41.9 (congenital absence, atresia and stenosis of duodenum, jejunum, ileum or small intestine, part unspecified), Q42.0-42.9 (congenital absence, atresia and stenosis of large intestine), Q43.1 (Hirschsprung’s disease), Q44.2 (bile duct atresia).

Genital abnormalities: Q54.0-54.9 (hypospadias).

Urinary system abnormalities: Q60.0-60.9 (renal agenesis and other reduction defects of kidney), Q61.0-61.9 (cystic kidney disease), Q64.1 (exstrophy of urinary bladder), Q64.2 (congenital posterior urethral valves).

Musculoskeletal system abnormalities: Q65.0-65.5 (congenital dislocation or subluxation of hip), Q66.0 (talipes equinovarus), Q66.1 (talipes calcaneovarus), Q66.4 (talipes calcaneovalgus), Q69.0-69.9 (polydactyly), Q71.0-73.9 (reduction defects of upper, lower and unspecified limb), Q75.0 (craniosynostosis), Q77.0-78.9 (osteochondrodysplasia with defects of growth of tubular bones and spine and other osteochondrodysplasias), Q79.0 (congenital diaphragmatic hernia), Q79.2-79.4 (exomphalos, gastroschisis, prune belly syndrome).

Chromosomal abnormalities: Q90.0-92.9 (Down’s, Edwards’ and Patau’s syndromes, and other trisomies and partial trisomies of the autosomes), Q93.1 (whole chromosome monosomy, mosaicism [mitotic nondisjunction]).

Note there are other malformations not appearing on this list that are also considered major by MACDP and/or EUROCAT, but these did not occur in any of the offspring born to Danish National Birth Cohort mothers.
