# Supplementary material for: Inflammatory Bowel Disease and Risk of Adverse Pregnancy Outcomes
Source: PLoS One. 2015 Jun 17;10(6):e0129567. doi: 10.1371/journal.pone.0129567 (PMC4471220; doi:10.1371/journal.pone.0129567)
Supplement: S3 File — (DOCX) [file pone.0129567.s003.docx]

**Pregnancy loss results including post-pregnancy IBD diagnoses in definition of IBD**

Relaxing our definition of an IBD-exposed pregnancy to include pregnancies in women diagnosed with IBD after the study pregnancy (up to December 2011) did not change our results appreciably. An additional 26 women with miscarriages were diagnosed with IBD (CD, 4; UC, 22) after the study pregnancy, along with two additional women with stillbirths (1 each with CD and UC). The HR for pregnancy loss associated with IBD overall was 0.61 (95% CI 0.33-1.14); the corresponding estimates for CD and UC were 0.68 (95% CI 0.25-1.81) and 0.58 (95% CI 0.26-1.29), respectively.
