# Supplementary material for: Inflammatory Bowel Disease and Risk of Adverse Pregnancy Outcomes
Source: PLoS One. 2015 Jun 17;10(6):e0129567. doi: 10.1371/journal.pone.0129567 (PMC4471220; doi:10.1371/journal.pone.0129567)
Supplement: S1 Table — (DOCX) [file pone.0129567.s004.docx]

**S1 Table. Complete Results for Specific Groups of Major Congenital Abnormalities**

|  | Risk Ratio (95% Confidence Interval)^a^ | | | | |
| --- | --- | --- | --- | --- | --- |
|  | Cleft palate ± cleft lip | Hip dislocation/subluxation | Talipes^b^ | Choanal atresia | Spina bifida |
| Inflammatory bowel disease overall | 1.73 (0.43, 6.97) | 1.58 (0.66, 3.81) | 1.51 (0.49, 4.71) | 51.4 (13.5, 195) | 8.40 (2.00, 35.2) |
| Crohn’s disease | 1.93 (0.27, 13.8) | 1.46 (0.37, 5.85) | 2.32(0.58, 9.29) | 125 (32.7, 478) | 20.2 (4.80, 84.9) |
| Ulcerative colitis | 1.57 (0.22, 11.2) | 1.67 (0.54, 5.18) | 0.89 (0.13, 6.33) | - | - |

^a^ Adjusted for maternal age (≤25, 26-30, 31-35, >35 years), parity (0, 1, ≥2), socioeconomic status (6 categories: master’s degree or higher and currently employed, or leader of a business with ≥10 employees; bachelor’s degree and currently employed, or leader of a business with <10 employees; skilled worker (completed vocational training with apprenticeship) and currently employed; unskilled worker or unemployed (short-term); current student; unemployed (long-term)), pre-pregnancy BMI (<20, 20-25, >25), and smoking (non-smoker, smoker), alcohol consumption (non-drinker, <1 drink/week, ≥1 drink/week) during pregnancy and folic acid use in the period from 4 weeks before conception to 8 weeks after conception (any use/no use).. **Exception**: estimates for choanal atresia and spina bifida are not adjusted for SES (model would not converge).

^b^ Talipes equinovarus (Q66.0), calcaneovarus (Q66.1), and calcaneovalgus (Q66.4) combined.
